# Unmasking the pathways to workplace incivility: A mediated moderation model of despotic leadership, workload stress, and distributive justice in higher education

**DOI:** 10.1371/journal.pone.0337687

**Published:** 2025-12-31

**Authors:** Muhammad Ahsan Ali, Haris Bin Khalid, Bilal Idrees, Zainab Naseer, Anum Sheraz

**Affiliations:** 1 Management Sciences, IBADAT International University, Islamabad, Pakistan; 2 Management Sciences, Namal University, Mianwali, Pakistan; 3 Management Sciences, Capital University Science and Technology CUST, Islamabad, Pakistan; IUB: The Islamia University of Bahawalpur Pakistan, PAKISTAN

## Abstract

The purpose of this study is to investigate how despotic leadership impacts workplace incivility through increased workload and to determine whether distributive justice moderates this relationship within selected higher education institutions in Rawalpindi and Islamabad. These two cities were specifically selected due to their significant representation of educational institutions, making them suitable samples for understanding dynamics within Pakistan’s higher education context. This study examines the relationship between despotic leadership (DL) and work place incivility (WPI) within the higher education sector of Rawalpindi and Islamabad. Specifically, it explores the mediating role of workload and the moderating role of distributive justice in this relationship. Grounded in the Conservation of Resources (COR) theory, this research extends existing literature by elucidating how resource depletion and accumulation shape employee behavior. A quantitative, cross-sectional survey-based methodology was utilized, collecting responses from 381 employees from higher education institutions located in Rawalpindi and Islamabad, with data analyses through IBM SPSS 27 and AMOS 22 using Confirmatory Factor Analysis (CFA), reliability tests, ANOVA, descriptive statistics, and correlation, and process macro direct effects, mediation, and moderation analyses to examine the proposed relationships. Indicates that despotic leadership significantly contributes to workplace incivility, primarily through increased workload. However, distributive justice serves as a mitigating factor, attenuating the adverse effects of workload on workplace incivility. The results confirm the mediating role of workload and the negative moderating influence of distributive justice. These insights underscore the necessity for organizational leadership to adopt more equitable and ethical management practices. Additionally, human resource policies should emphasize fairness and actively address complaints related to unfair treatment. The study posits that maintaining fairness in workload distribution, enhancing hiring practices to deter the emergence of despotic leaders, and establishing secure mechanisms for reporting grievances are critical steps for organizations seeking to curb workplace incivility. It underscores the centrality of distributive justice in mitigating negative interpersonal dynamics and fostering a more positive organizational climate. Moreover, initiatives such as impartial investigations and civility training programs are identified as pivotal in strengthening workplace relationships and preventing the escalation of retaliatory behaviours that contribute to a spiraling effect of incivility. Our study is limited by its focus on higher education institutions in Islamabad and Rawalpindi, suggesting the need for future research across broader sectors, cities, and global contexts. This research extends prior work in organisational behaviour and leadership studies, particularly by building upon the Conservation of Resources (COR) theory and the workplace incivility literature. It empirically examines the effect of despotic leadership on workplace incivility, highlighting workload as a mediating mechanism and distributive justice as a moderating force. By focusing on the higher education context, the study addresses a significant gap, providing a nuanced understanding of how leadership dynamics and perceptions of fairness jointly influence patterns of incivility through a mediated moderation framework.

## Introduction

Leadership is key and plays a significant role in shaping organizational success [1]. Effective leadership promotes a process of beneficial social significance, which maximizes employee effort and dedication, benefiting both the organization and its employees [2]. Leaders must be able to influence subordinates to work towards the organization’s objectives. This field has historically been idealized, focusing on the positive impact of leadership for followers and organizations while ignoring/neglecting its dark sides [3]. The present research has begun to examine the potential negative effects of leadership [4].

Leadership style plays a crucial role in shaping employee performance, particularly in Eastern countries where the” rule by man” approach, where authority is more personalized and central to decision-making, is more prevalent. Much of the research in these regions focuses on how good leadership can help organizations succeed [5]. However, there is less research on the negative side of leadership [6], even though these negative behaviors are often more common in Eastern countries because of the strong power distance in these cultures [7]. Negative leadership styles such as abusive, toxic, autocratic, and exploitative leadership have been identified [8], with despotic leadership being the most harmful. Despotic leadership involves leaders who demand complete obedience, hold total power over their subordinates, and mistreat them [9]. Although its harmful, despotic leadership is still under researched, particularly in management and psychology, especially in countries with high power distance like Pakistan [10].

Despotic leaders are often self-centred and unethical, putting their own interests ahead of their employees’ well-being. Such leaders may manipulate their workers, lie, and even engage in dishonest behavior [5]. The financial costs of despotic leadership are huge, with estimates showing it costs $23.8 billion each year and affects 13.6% of workers in the United States [11]. This shows that despotic leaders care more about their own gain than about their employees or organizations [12,13]. In countries like Pakistan, where power distance and collectivism are high, employees often feel they must obey their leaders without question, and cultural norms accept power differences. Many workers in Pakistan also feel forced to put up with these conditions due to poverty and high unemployment. Due to these cultural values, employees in Eastern countries may tolerate despotic leadership [14]. This shows that it’s important to understand the harmful effects of despotic leadership in countries like Pakistan [15]. This study looks at how despotic leadership impacts workplace incivility in the higher education sector in Pakistan’s Islamabad-Rawalpindi region.

Despotic leadership is characterized by authoritarianism and dominating behavior, which creates a stressful work environment by increasing pressure, micromanagement, and unrealistic demands. It is a style of leadership in which the leader is strict and overly controlling and doesn’t care about employees’ resources [16–19]. These traits can potentially harm workers, increasing the risk of information distortion, manipulation, and other types of corruption within organizations [20].

The notion of” despotic leadership” is distinct since it only describes a particularly harmful form and pattern of exploitative, authoritarian, and dominating behavior [21,22]. Workers who are subjected to despotic leadership frequently feel high levels of stress, discontent, and emotional strain; this eventually results in emotional exhaustion, a crucial element of burnout [23].

This leadership style, while clearly linked to negative employee outcomes, triggers complex behavioral responses. As Maslach, Schaufeli [23] notes, three things make up an attitude: conduct, affect, and cognition. Cognition refers to mental processes based on previous experiences, affect reflects the psychological aspect, and behavioral refers to the inclination to act in a specific way. These components interact with workplace stimuli, and together they shape employee responses, making it essential to consider the affective, cognitive, and behavioral aspects of attitude when analysing how despotic leadership influences employee behavior [24].

Workplace incivility is the result of stress and frustration from excessive workloads. It is a form of low-level misbehavior that breaks the norms of mutual respect [25]. Incivility can come from leaders, colleagues and subordinates, and it easily spreads throughout the organization, reducing trust, teamwork, and employee morale, ultimately affecting workplace environment [26,27]. Workplace incivility is a type of low-intensity deviant behavior that violates norms of mutual respect.

It is characterized by subtle, often ambiguous, actions that may or may not be intended to harm others. Examples include behaviors such as rudeness, disrespect, ignoring or excluding coworkers, or undermining colleagues’ contributions. Though less overt than bullying or harassment, Incivility can have catastrophic consequences for both individuals and organizations. Incivility can lead to job dissatisfaction, emotional exhaustion, and eventually increased employee turnover [28]. Furthermore, workplace incivility can lead to more serious types of workplace negativity, which contributes to a toxic work environment. This form of deviant behavior is often cyclical, with incivility begetting more incivility as employees retaliate or react negatively to being treated disrespectfully [29]. Workplace incivility refers to low-level behaviors that can negatively impact others, even if there’s no clear intention to cause harm. These can include things like making rude or dismissive comments, ignoring someone’s ideas, giving them the silent treatment, or even yelling or insulting them [30]. While similar to microaggressions and discrimination, incivility specifically involves breaking the unwritten rules of respect and politeness in the workplace [31]. These behaviors can come from different people, including supervisors, coworkers, or clients [32]. Studies reveal almost all employees in North America experience some form of workplace incivility, which highlights how common and concerning the issue is [27].

Although workplace incivility is often seen as mild mistreatment, research over the years has shown that it can have serious long-term effects on employees’ health and well-being [33–35]. The impact of incivility, however, can vary depending on individual and workplace factors, meaning that not all employees be affected in the same way [36]. Many studies have explored how even small disrespectful actions can lead to larger, more complex consequences for both employees and organizations [37–39]. This research underlines the significance of preventing work place incivility to protect the resources of the employees and provide good work environment.

Under despotic form of leadership (which is associated with authoritarian and controlling behaviour), the employees’ workload is frequently increased because of unrealistic expectations and excess requirements made upon the employees. Such leaders are result oriented and not concerned with employees’ well-being, thus forcing the employees to work over capacity. Consequently, the employees become overwhelmed and find it hard to cope with their task, creating more stress, frustrations, and negative feelings that might end up shaping the workplace.

### Incivility

Workload refers to the tasks and responsibilities assigned to an employee. When workload becomes excessive or unfair, it can negatively impact, behaviors, causing stress, fatigue, emotional strain, and health issues such as headaches and irritability. It may also lead to workplace incivility, reduced productivity, and increased employee turnover [40]. Workload means the number of tasks and activities that companies ask employees to complete within certain time limits [41]. In schools and universities, this can include teaching students, organizing activities, attending meetings, and doing research. From an organization’s point of view, giving workers a set amount of tasks can reduce laziness, which helps to increase productivity and teamwork [42].

However, if workers have too many tasks to complete, it can harm their physical and mental health [42]. There is still debate about how much workload is acceptable to improve productivity and teamwork without causing harm. A heavy workload can lead to incivility in the workplace. When employees are overloaded with tasks, it can drain their resources and result in negative behaviors [43]. Work-related stress occurs when employees find it difficult to cope with the demands placed on them [44]. One major factor that affects employee behavior is the unequal distribution of work, which can encourage incivility among colleagues [45].

Additionally, a heavy workload can lead to fatigue, which negatively impacts the work environment and the overall culture within the organization [46]. The workload is often identified as a situational factor that influences workplace behavior, especially workplace incivility [47]. A heavy workload is typically seen as a hindrance, and when employees feel unable to modify or control this workload, negative emotions can build up and potentially lead to aggressive behaviors toward coworkers [25]. Workplace incivility can manifest as rude or dismissive behavior, which is often a response to the overwhelming pressures caused by heavy workloads. However, this relationship is not straightforward. Some studies suggest that, under certain conditions, employees may view heavy workloads as challenges rather than obstacles, which can lead to positive outcomes [48]. Additionally, studies show that high job demands could force workers to dedicate all of their energy and time to their primary responsibilities, which might reduce their ability to participate in low-intensity aggressive behaviors like rudeness [49]. Nonetheless, when combined with stresses like job unhappiness and work tiredness, hefty workloads can result in bad behaviors like rule-breaking, stealing, or quitting the company. Feelings of distributive injustice, where employees perceive an unfair distribution of workload or resources, may amplify these negative behaviors, leading to increased frustration and workplace incivility [50].

Distributive justice refers to employees’ perceptions of fairness regarding workplace outcomes like salary, workload, promotions, and other benefits [51]. Adams [52] suggests that employees assess fairness by comparing their inputs (e.g., effort) to the outputs (e.g., rewards) they receive. Perceived imbalances can lead to negative outcomes, such as emotional exhaustion and withdrawal [53]. While distributive justice is important, perceived fairness in processes and interpersonal treatment is more strongly linked to employee responses to supervisors and organizations.

When employees perceive injustice, they may engage in unethical behaviors like retaliation or rule violations. Distributive justice, tied to fair rewards, influences such actions as employees attempt to restore perceived imbalances in input and output.

Employees who experience distributive justice attain valuable protection against stressful situations caused by despotic leadership and large workloads. Employees who perceive fair distributions of rewards and resources handle stress in a constructive manner while injustice perceptions will amplify stress and lead staff to disengage and drop performance and escalate workplace incivility (Tepper, 2000). Unfair distributions of authority alongside demanding workloads will worsen employee mistreatment which in turn causes workers to respond with retaliation while reducing their level of teamwork. When alleged favoritism prevails in organizational management it harms both culture quality and productivity while also decreasing employee motivation and retention levels. The combination of despotic leadership with excessive workloads serves to strengthen incivility but distributive justice either helps decrease or increase these combined effects. An understanding of these interdependent workplace elements enables the development of reduction strategies for destructive leadership problems and uncivil conduct.

This study fills numerous contextual and theoretical gaps in the literature on despotic leadership and workplace incivility that previous studies have not addressed. In particular, there is a need to explore how despotic leadership affects employees’ behavior. Leadership is a crucial part of employees’ effectiveness in achieving organizational goals. Effective/Good leadership helps employees feel in Control and treated fairly; on the contrary, despotic leadership takes away that sense of Control and justice, which can lead to negative consequences.

According to research, despotic leadership causes people to feel dominated and controlled, resulting in a lack of justice. This often leads to increased frustration, stress, and negative emotions; if left unchecked, these types of issues turn into workplace issues like incivility in the workplace environment. These negative emotions can escalate into workplace incivility and even result in employees quitting their jobs due to distress [11,54,55]. All of these variables have been previously documented in the literature, yet they are not assembled in a single conceptual model. The current study modeled all of these variables by examining the impact of despotic leadership on workplace incivility, with workload as a mediating factor and distributive Justice as a moderator. Despite extensive research on despotic leadership and its negative impact on workplace incivility, several critical gaps remain.

First, while numerous studies have explored the direct effect of despotic leadership on workplace incivility, the mediating role of workload in this relationship has not been adequately addressed. Excessive workload, which can create difficulties in employee well-being and performance, can significantly impact the extent to which despotic leadership leads to workplace incivility [56].

Second, it has not been adequately investigated how distributive justice moderates the relationship between workload, workplace incivility, and despotic leadership. Research indicates that perceptions of fairness in resource distribution can significantly impact employee behavior, potentially reducing the negative effects of despotic leadership [57]. Understanding these relationships is crucial for promoting a positive workplace environment. Furthermore, research in the Pakistani higher education sector in the context of leadership is minimal. Accordingly, there is a pressing need to expand research in the sector by examining the negative impact of despotic leadership within Pakistan’s higher education sector. This is particularly relevant for higher education, as the study examines the negative effects of despotic leadership. It highlights how such leadership can create an authoritarian/dominating atmosphere and lead to workplace incivility, ultimately increasing employee turnover intentions [58].

Recent reports highlight a rising prevalence of workplace incivility in higher education, particularly in Pakistan. Anwaar, Yusof [59] found that 71% of university faculty have encountered incivility, underscoring the severity of this issue. In recent years, despotic supervision has emerged as a critical concern within Pakistan’s higher education sector, adversely affecting employees. Characterized by authoritarian and hostile behaviours, this leadership style fosters a tense work environment, leading to negative outcomes such as increased workload, diminished perceptions of distributive justice, and heightened workplace incivility. The growing prevalence of despotic leadership has prompted increased scholarly attention to its role in exacerbating workplace incivility. According to the Higher Education Commission (HEC), Islamabad and Rawalpindi collectively house 36 universities, employing approximately 98,452 faculty members of whom 37,209 are female and 61,243 are male. Reports suggest that many employees in these institutions experience elements of despotic supervision, contributing to withdrawal behaviours, elevated stress levels, and increased absenteeism. These factors ultimately result in reduced efficiency, low morale, heightened anxiety, and unethical conduct. Given the profound implications of despotic supervision for professionals in Pakistan’s higher education sector, it is imperative to examine its impact on workplace incivility. This study specifically investigates the mediating role of workload and the moderating role of distributive justice in this relationship. By analysing these dynamics, the research provides a strategic framework aimed at mitigating workplace incivility and fostering a more constructive and productive work environment. The current study intends to address two main research questions.

RQ 1. Does the despotic leadership affect workplace Incivility.?

RQ 2. Does workload mediate the relationship between despotic leadership and workplace incivility.?

RQ 3. Does distributive justice moderate the relationship between workload and workplace Incivility.?

The Conversation of Resource (COR) theory supports all variables of the proposed research. The COR theory covers all the variables properly and provides both direct and indirect links between various variables. This study examines the impact of despotic leadership on workplace incivility, focusing on the mediating role of workload and the moderating role of distributive justice. The COR theory helps explain the relationships between despotic leadership, workload, distributive justice, and workplace incivility, providing a clear pathway for understanding how resource depletion impacts employee behavior and organizational outcomes. The COR (Conservation of Resources) theory, first proposed by [60]. is a theory of stress that explains the implications of stress and its management in life. In this theory, resources are things that employees value, like objects, conditions, or qualities that help them succeed. These resources can be psychological, physical, personal, social, or material [60,61]. Stress happens when these resources are threatened, lost, or used up, especially in difficult work environments where leadership adds extra pressure. As a result, people try to get, protect, and keep the resources they need to handle stress.

If we relate the COR theory to the proposed research model, despotic leadership, characterized by authoritarianism and dominating behavior, often forces employees to handle excessive or irrelevant tasks, increasing their workload. This leads to a reduced perception of distributive justice due to the leader’s despotic behavior, ultimately resulting in the depletion of employees’ psychological resources. When psychological resources are depleted, employees may show bad behavior in the workplace, which, as a result, leads to workplace incivility. In the absence of distributive justice, despotic leadership increases workplace incivility. When employees perceive an unfair distribution of resources and workloads, the negative effects of despotic leadership increase, leading to increased stress and strengthened workplace incivility. Thus, the current theory is an overarching theory for the proposed research model.

**Fig 1:**
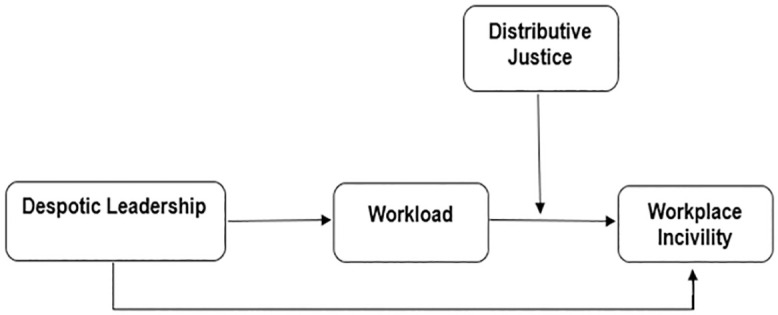
Research model.

This research offers information for organizations to support employees better. Knowing these dynamics can increase job performance, reduce turnover, and decrease workplace incivility in the face of despotic leadership and excessive workload. The conceptual framework of this study investigates the impact of despotic leader ship on workplace incivility, with workload as a mediator and distributive justice as a moderator. So, this study fills these gaps by examining the mediated moderation model with the impact of despotic leadership on workplace incivility via workload and distributive Justice used to reduce the negative effect of despotic leadership on workload in the higher Education Sector Islamabad/Rawalpindi. (Fig 1 Research Model).

## Theorization and hypothesis development

### Despotic leadership and workplace incivility

Despotic leadership is marked by authoritarian, self-serving behavior where leaders prioritize their interests, demand unquestioning obedience, and disregard employee well-being [11,55]. Such leaders are emotionally exploitative, lacking empathy and engaging in irrational, unfair decision-making [62,63]. This oppressive climate reduces job satisfaction, organizational commitment, and fosters toxic workplace behaviors [22,53,64]. Under despotic regimes, employees often face emotional distress and may resort to incivility or bullying as a coping mechanism [65], perpetuating a cycle of toxicity [26].

Workplace incivility characterized by low-intensity disrespectful behavior like sarcasm, exclusion, and lack of cooperation, thrives under such leadership, eroding organizational culture [66]. Despotic leaders further worsen this by exploiting power distance, displaying favoritism, and undermining collaboration [67]. Research shows despotic leadership stifles creativity, suppresses independent thought, and reduces innovation [68], while neglecting employees’ emotional states [69]. Under the theoretical lens of Conservation of Resources (COR) theory [60,70] when resources like energy, support, and control are depleted by despotic leadership, stress ensues [71]. This depletion fuels burnout and incivility [8], especially in ethically oriented employees or newcomers [72,73]. Defensive behaviors like bullying or workplace deviance then emerge, exacerbating the toxic environment [30,35].

- H1: Despotic leadership has a positive and significant impact on workplace incivility.

### Despotic leadership and workload

Despotic leadership, marked by authoritarianism and self-interest, demands excessive obedience and often disregards subordinates’ well-being [11]. This leadership style creates a toxic environment that harms morale and organizational performance [74]. A key consequence is increased workload, negatively impacting employee well-being, both physically (e.g., headaches) and psychologically (e.g., anxiety, fatigue) [75]. Despotic leaders impose high job demands without regard for employee input or autonomy, causing stress and burnout [76]. Such leaders increase pressure, leading to job dissatisfaction and turnover intentions [77].

These Leaders create workload pressure for an employee. Workload pressure, defined as overwhelming demands consuming time and energy [78], often leads to burnout, emotional exhaustion, and reduced productivity [79]. Employees often view despotic leaders as hostile and exploitative, involving verbal and non-verbal abuse [53]. Around 13.6% of U.S. workers experience such leadership, costing firms billions [21]. It’s linked to depression, anxiety, emotional exhaustion, and burnout [80]. As a result, it is recognized as a serious organizational concern [81].

Despotic leadership also increases workplace incivility by overburdening employees, eroding their ability to remain civil [82,83]. Under the umbrella of Conservation of Resources theory [60], stress arises when valuable resources (time, energy) are threatened. Employees under such leadership may retaliate or disengage as a coping mechanism. High job demands and leadership hostility drive turnover intentions [84]. Without support, employees feel trapped and burned out, often choosing to leave for healthier environments [85]. Despotic leadership thus fosters a hostile climate that fuels workload stress, burnout, incivility, and reduced organizational commitment.

- H2: Despotic leadership has a positive and significant impact on workload.

### Workload and workplace incivility

Workload refers to the tasks and responsibilities employees must complete within a specific time [41]. In academia, it includes teaching, events, meetings, and research. A reasonable workload can enhance performance and teamwork [42], but excessive demands often result in stress and workplace incivility [86,87]. Employees under high workload may feel overwhelmed, leading to frustration and uncivil behaviors, especially when deadlines or expectations feel unmanageable [88]. Despite some beliefs that more workload boosts productivity, studies also link it to anxiety, burnout, and incivility [89–91]. Stress from excessive workload often results in irritability and low cooperation, fueling uncivil acts [92].

Workplace Incivility is linked to reduced job satisfaction, poor atmosphere, low managerial support, and diminished control [39,93–98]. While job demands correlate with incivility [99]. the direct link between workload and incivility remains inconsistent [100]. This inconsistency may stem from how workload is measured. Hasson, Okazawa [101] argue that unidimensional tools may miss crucial aspects. A multidimensional approach could better reveal its impact on incivility. Occupational health guidelines Corin, Pousette [102] emphasize “unhealthy workload,” highlighting its relevance to well-being. Yet, gaps remain in clearly associating workload with incivility.

Burnout significantly differs between employees who experience incivility and those who do not, suggesting workload is linked to negative outcomes, even if indirectly. Conservation of Resources theory explains that depleted psychological resources can lead employees to justify incivility [103–105]. Employees under heavy stress may engage in rule-breaking or rudeness as coping mechanisms [106–108]. When combined with psychological entitlement, such behavior is exacerbated [109]. Employees who feel overburdened and unrecognized may react negatively, especially if they believe they deserve better treatment [110]. This can manifest in subtle or overt incivility, justified as retaliation. Such patterns can lead to a toxic work environment, reducing morale, productivity, and employee commitment (C. H. Liu et al., 2024). Leadership plays a key role in addressing these issues through fair task allocation, recognition, and supportive communication.

Fostering respect and empathy while managing workload expectations can reduce stress and mitigate uncivil behavior. When employees feel supported, they are less likely to act out. Thus, workload especially when paired with psychological entitlement can be a critical driver of workplace incivility.

- H3: Workload has a positive and significant impact on workplace incivility.

### Workload mediates between despotic leadership and workplace incivility

Workload mediates despotic leadership and workplace incivility relationship [19,98]. Despotic leadership, marked by controlling behaviors, creates a high-pressure work environment, leading to excessive workload, which depletes employees’ resources and causes psychological strain [60]. Despotic leadership also violates the psychological contract, which includes expectations of respect and fair treatment [111]. When these expectations are unmet, employees perceive workload increases as unjust, leading to emotional exhaustion and incivility [8]. Employees cope with high demands by engaging in uncivil behaviors [112]. Over time, high workloads and despotic leadership lead to emotional exhaustion, anxiety, and disengagement, which contribute to incivility [5,113].

Workload acts as a mediator, exacerbating the effects of despotic leadership and incivility. The combined pressure depletes personal resources, leading to workplace incivility, such as aggression or withdrawal, which harms team dynamics and morale [114]. This creates a toxic work culture, reducing productivity and satisfaction. Organizations should address workload balance and support employees to reduce the impact of despotic leadership and incivility. The Conservation of Resources (COR) theory suggests that employees seek to protect their resources when facing stressors like harsh leadership [115]. High workloads resulting from despotic leadership increase emotional distress and contribute to workplace incivility [116,117].

- H4: Workload mediates the relationship between despotic leadership and workplace incivility.

### Distributive Justice moderates the relationship between Workload and Workplace Incivility

Distributive justice refers to employees’ perceptions of fairness in the distribution of outcomes such as workload, salary, and promotions [51]. When employees perceive fairness in these areas, they feel valued and satisfied, but perceptions of inequity can lead to frustration and conflict [118]. Distributive justice influences attitudes and behaviors, with unfair treatment linked to negative outcomes like workplace incivility [27,119]. Incivility is often a response to perceived injustice, especially in workload distribution [25,120]. Distributive justice moderates the relationship between workload and workplace incivility. When employees perceive fair workload distribution, they feel valued and manage stress better, reducing incivility [121]. In contrast, perceived inequities lead to stress and incivility. COR theory [60] suggests that distributive justice helps conserve resources, buffering the negative effects of high workloads. High distributive justice reduces stress, enabling employees to cope and preventing emotional exhaustion and uncivil behavior [122].

Employees who perceive fairness seek support and use stress management strategies to handle workloads [123]. Prioritizing distributive justice through transparent decision-making and equitable resource allocation fosters a supportive environment, increasing job satisfaction and reducing incivility [124]. Training managers to ensure fairness in workload distribution also enhances employee trust and reduces dissatisfaction [125]. Distributive justice improves mental health, reducing conflict and fostering a productive work culture [126].

- H5: Distributive Justice moderates the relationship between workload and workplace incivility; such that the relationship will be weakened when distributive Justice is high and vice versa.

## Methods

This study adopts a positivist research philosophy, emphasizing that true knowledge arises from observable and measurable data Saunders [127]. A deductive approach was used, with hypotheses derived from theory and tested through empirical evidence. A quantitative, cross-sectional research design was employed to examine the relationships between despotic leadership, workplace incivility, workload, and distributive justice in higher education institutions in Islamabad and Rawalpindi.These two cities were specifically selected due to their significant representation of educational institutions, making them suitable samples for understanding dynamics within Pakistan’s higher education context. The unit of analysis comprised individual faculty members, HODs, and Deans. Data were collected in natural, non-contrived settings with minimal researcher interference. The population included academic staff from 36 public and private universities in the selected regions. Using Morgan [128] table, a sample size of 381 was determined based on a 95% confidence level and a 5% margin of error. Convenience sampling was applied due to time and access constraints. A total of 500 structured questionnaires were distributed; 119 were unusable, leaving 381 valid responses and a response rate of 83.3%. According to [129]. such a high response rate reflects the study’s relevance and rigour, indicating that respondents considered the topic significant. Ethical standards were maintained through informed consent, voluntary participation, and assurance of anonymity and confidentiality [130]. Data analysis was conducted using SPSS 27 and included reliability analysis, descriptive statistics, ANOVA, correlation, regression, and mediation and moderation analyses.

Consent was obtained from all participants before data collection. Data were collected using a self-administered questionnaire, which included a written consent statement on the first page. Participants were fully informed about the purpose of the study, the voluntary nature of their participation, the anonymity of their responses, and their right to withdraw from the study at any time without any consequences. The participants were employed in the higher education sector of Islamabad, and Rawalpindi was included in the study, with no minors involved. Additionally, participants were assured that all information provided would be kept strictly confidential, that no organisational data would be disclosed, and that individual responses would not be identifiable in any published reports. These measures ensured adherence to ethical guidelines for research involving human participants. The entire data collection process adhered strictly to the established ethical guidelines for research involving human participants. Notably, this research did not involve any experiments or clinical trials with humans or animals, nor did the questionnaire require any sensitive information.

### Instruments

Data collection used a structured questionnaire. The instrument was divided into four major sections: workplace incivility (WPI), workload (WL), despotic leadership (DL), and distributive justice (DJ). Instruments used in the study have been adopted. Table 1 presents detailed information about each instrument utilized in the study.

**Table 1:** Measurements and scales.

Variables	Developed By	Items
**Despotic Leadership**	De Hoogh and Den Hartog (2008)	6
**Workload**	Hart (1988)	6
**Distributive Justice**	Niehoff and Moorman (1993)	5
**Workplace Incivility**	Cortina et al. (2001)	7

**Despotic Leadership (DL):** Six items developed by De Hoogh and Den Hartog [64] for measuring Despotic Leadership were used. The sample item is ‘My leader is punitive, no pity or compassion.’

**Workload (WL):** Assessment of workload was done with six items from the NASA-TLX (Task Load Index) developed by [131]. One sample item, of course, is’The tasks assigned by my leader challenge my cognitive or mental abilities.’

**Distributive Justice (DJ):** Distributive Justice was measured using five items developed by Niehoff and Moorman [132], A sample item is,” My work schedule is fair.”

**Workplace Incivility (WPI):** Workplace Incivility (WPI): Seven items developed by Cortina, Kabat-Farr [36] were used to evaluate Workplace Incivility. An item “My colleagues always put me down or they are condescending to me.”

This followed the use of a. 5-point Likert scale from’Strongly Disagree’ to’Strongly Agree’ which allowed respondents to assert a degree of agreement or disagreement with each one of the items presented on the questionnaire.

## Results and analysis

### CFA confirmatory factor analysis

IBM Amos 24 was used to check if the model fits the data. It helps us decide whether the model is good enough for further analysis. First, we need to confirm that the constructs are valid, and then we can test the hypotheses. We looked at fit indices like chi-square, IFI, TLI, CFI, and RMSEA, which tell us whether the model fits well.

Gaskin (2016) explains that for a model to fit well, both the Comparative Fit Index (CFI) and Incremental Fit Index (IFI) should be higher than 0.90. These values compare the proposed model to a simple one, and higher numbers show a better fit. For the Tucker-Lewis Index (TLI), it should also be above 0.90, with 0.95 being ideal. The Chi-square test checks how well the model matches the data, and a value below 2 or 3 is good. The Root Mean Square Error of Approximation (RMSEA) shows how close the model is too perfect, with values below 0.08 considered good, and below 0.05 even better. In this analysis Table 2 presents the model fit was acceptable and ready for further analysis without removing any item. Overall, the model fit is now good, and we can test if the different paths are significant.

**Table 2:** Confirmatory factor analysis and measurement model validation.

	CMIN/ DF	IFI	TLI	CFI	RMSEA
**Four Factor Model**	1.559	0.913	0.912	0.903	0.041

#### Descriptive statistics.

Before examining the correlations, a one-way ANOVA was conducted to assess the impact of demographic variables (gender, age, education, and experience) on the dependent variable, workplace incivility. All demographic variables were statistically non-significant (p > 0.05), indicating no meaningful influence on the outcome variable. Therefore, these factors were excluded from further analysis and did not require control, as they had no impact on the study’s results.

Table 3 presents the internal consistency reliabilities (Cronbach’s alpha), descriptive statistics (means and standard deviations), and Pearson correlation coefficients among the study variables. The correlation analysis (Table X) reveals a strong positive relationship between despotic leadership (IV) and workplace incivility (DV) (r = 0.834**, p < 0.01). Workload (Med) mediates this relationship, correlating positively with both despotic leadership (r = 0.526**, p < 0.01) and workplace incivility. Distributive justice (Mod) moderates the relationship, showing negative correlations with despotic leadership (r = −0.495**, p < 0.01) and workplace incivility (r = −0.548**, p < 0.01), indicating its mitigating effect. In conclusion, the results indicate that despotic leadership has a positive and significant impact on increasing workload and decreasing distributive justice. These factors, in combination, contribute to higher levels of workplace incivility.

**Table 3:** Descriptive statistics, reliability, and correlation matrix.

Variables	(α)	Min	Max	Mean	SD	DL	WL	DJ	WPI
**Despotic Leadership**	0.848	1.40	5.00	4.17	0.71	1			
**Workload**	0.803	1.50	4.83	4.14	0.59	.526**	1		
**Distributive Justice**	0.808	1.00	5.00	1.89	0.78	−.495**	−.617**	1	
**Workplace Incivility**	0.816	1.71	5.00	4.03	0.69	.834**	.587**	−.548**	1

Note(s): *N* = 381; **p < 0.01 (2-tailed); *SD* = Standard Deviation; *Min.* = Minimum; *Max.* = Maximum.

#### Hypothesis testing.

**Direct and indirect hypotheses:** To test the direct and indirect hypotheses, Hayes’ PROCESS Macro (2013) was used, specifically Model 4 in IBM SPSS 27. The mediation analysis applied the bootstrapping procedure with 5,000 resamples, following the guidelines of Preacher and Hayes (2004). Table 3 result supported to hypothesis 1,2,3 and 4. The findings reported in Table 4 provide robust support for the hypothesized relationships. Specifically, Hypothesis 1 is substantiated by a significant and positive association between despotic leadership and workplace incivility (*effect* β =* 0.80, SE = 0.027, p < 0.001*), suggesting that higher levels of despotic leadership correspond with increased incidences of incivility among employees. Hypothesis 2, positing that despotic leadership elevates employee workload, is similarly supported (effect *β = 0.439, SE = 0.036 p < 0.001*), indicating that despotic behaviors intensify employees perceived work demands. Further, Hypothesis 3, which argues that workload exacerbates workplace incivility, is confirmed (*effect* β =* 0.68, SE = 0.048 p < 0.001*), highlighting the role of excessive workload in fueling negative interpersonal dynamics. Crucially, Hypothesis 4 indirect effect demonstrates that workload serves as a significant mediator in the relationship between despotic leadership and workplace incivility (*indirect effect* β =* 0.1046, SE = 0. 0261 p < 0.001, LLCI 95% = 0.058 ULCI 95% = 0.159*), underscoring the mechanism through which authoritarian leadership styles indirectly foster uncivil behaviors via increased job demands. Collectively, these results illuminate the spiraling effect by which despotic leadership engenders a toxic work environment characterized by escalating incivility.

**Table 4:** Direct and indirect effect.

Direct Effect	B	S. E	P	LLCI	ULCI
Despotic Leadership *→ WPI*	0.809	0.027	0.00	0.755	0.863
Despotic Leadership *→ Workload*	0.439	0.036	0.00	0.367	0.510
Workload →* *WPI	0.682	0.048	0.00	0.367	0.510
**Indirect Effect**	**B**	**S. E**	**P**	**LLCI**	**ULCI**
Despotic Leadership *→ *Workload →* *Workplace Incivility	0.1046	0.0261	0.00	0.0583	0.1595

Note: *N* = 381; Bootstrap sample size = 5000; CI = Confidence Interval; LL = Lower Limit; UL = Upper Limit; **p < 0.000.

### Mediated moderation analysis

To test the moderation model, Model 14 of PROCESS Macro was employed. Hypothesis 3 proposed that Distributive Justice moderates the relationship between Workload and Workplace Incivility**;** such that the relationship will be weakened when distributive Justice is high and vice versa. The results presented in Table 5 provided empirical support for this hypothesis, as indicated by the significant interaction term (*effect* β = *−0.1371, SE = 0.041, p < 0.01, LLCI −.2187, ULCI −.055*).

**Table 5:** Moderation effect.

(Moderator Variable: Distributive Justice)	B	S. E	P	LLCI	ULCI
Int-term WL*DJ	−0.1371	0.041	0.001	−0.2187	−0.055

N = 381, Workload x Distributive Justice = int-term

Moreover, to examine the mediated moderation model, the results of the conditional indirect path are provided with (−1SD, + 1SD) are provided in Table 6. Also, Table 7 showed the index of mediated moderation in which both the lower level and upper-level confidence interval (*ULCI −.1267 ULCI −.0022*) have same sign and no zero is present between them. This shows that distributive justice moderated the indirect of despotic leadership on workplace incivility through workload, providing support to the full model.

**Table 6:** Conditional indirect effect of X on Y at values of the moderator.

Mediator	Distributive Justice	Indirect effect	Boot SE	LLCI	ULCI
Conditional indirect effects at M + 1SD					
**Workload**	4000	0.1310	0.0689	0.2325	0.2086
**Workload**	6000	0.1190	0.0325	0.0629	0.1894
**Workload**	6000	0.0588	0. 0330	−0.0013	0.1268

Note: N = 381. Bootstrap sample size = 5000. The range of values represents an abbreviated version of the output produced by the SPSS macro, LL Lower limit; CI Confidence interval; UL Upper limit CI confidence.

**Table 7:** Index of mediated moderation.

Mediator	Index	Boot SE	LLCI	ULCI
**Workload**	−.0601	.0313	−.1267	−.0022

**Fig 2:**
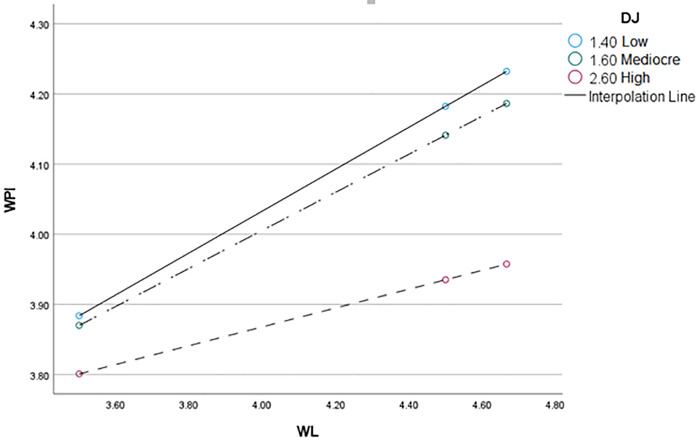
Graph.

To further validate the moderation effect, an interaction plot was generated and is presented in Fig 2. The graph illustrates the buffering role of Distributive Justice in the relationship between Workload and Workplace Incivility.

Workplace incivility emerges not in isolation but as a consequence of underlying organizational dynamics. The findings suggest that as workload (WL) increases, workplace incivility (WPI) escalates, reinforcing the notion that resource strain fosters behavioural deviations. Yet, this relationship is neither uniform nor absolute. Distributive Justice (DJ) functions as a critical moderator, altering the trajectory of this interaction. When DJ is low (1.40), the strain imposed by workload amplifies uncivil behaviours, as employees perceive inequitable resource allocation. In contrast, higher DJ (2.60) tempers this effect, diffusing the stress-induced spillover into workplace interactions. This pattern underscores the insidious nature of incivility subtle yet pervasive shaped by the interplay of structural fairness and job demands. Left unchecked, incivility may propagate, reinforcing a cycle of disengagement and conflict. However, where fairness prevails, workplace interactions stabilize, mitigating the corrosive effects of escalating workload pressures.

## Discussion

Our findings support the first hypothesis of our study, which states that” Despotic leadership has a positive and significant impact on workplace incivility.” This hypothesis aligns with previous studies showing how despotic leadership affects workplace incivility. Specifically, we used the Conservation of Resources (COR) theory Hobfoll [60,61] to explain the direct link between despotic leadership and workplace incivility.

Liu, Zhang [133] Under despotic leadership characterized by authoritarianism and dominating behavior, leaders often force employees to handle excessive or irrelevant tasks, increasing their workload. According to [6,7]. This results in a lower perception of distributive justice due to the leader’s despotic behavior, which leads to the depletion of employees’ psychological resources. When psychological resources are depleted, employees may display destructive workplace behavior, ultimately leading to workplace incivility incivility. This link between the dark side of leadership and workplace incivility is well-documented in the literature. For instance, O’Donoghue, Conway [134] discovered that despotic supervision is detrimental to the work behaviour. Alex Praveen Raj, Nelson [135] also identified a positive connection between despotic leadership and workplace incivility.

Most studies report a positive association between despotic leadership and incivility, research [11], has found a weaker relationship in collectivist cultures, suggesting that the cultural context may moderate the strength of this link. Our findings from the higher education sector in Islamabad/Rawalpindi contribute to this debate by highlighting that even in environments where hierarchical relationships are often normalised, despotic leadership still significantly increases incivility, perhaps due to the increased professional expectations in academia [7].

Nauman, Zheng [5] pointed out the need to better understand the mechanisms through which despotic leadership triggers bullying behavior among employees [136], which ultimately impacts the overall workplace environment [137]. Despotic leaders tend to create a climate of intimidation, fostering hostility among followers. Their negative actions, including domination and authoritative behavior, distract workers from their goals and create a toxic workplace atmosphere [7]. This goes along with our findings and further confirms the negative impact of despotic leadership on workplace incivility.

Hypothesis 1 posits that despotic leadership has a positive impact on workplace incivility. This finding aligns with [138]. who suggested that workplace incivility positively influences despotic leadership. Our research is extension to [139] research as we look at the workplace incivility despotic leadership relationship in the higher education sector in Isb/Rwp that has authoritarian elements in the workplace of the academic environment.

Although despotic leadership and incivility at the workplace have been broadly researched across the world in the last two decades [39]. This study thus investigates the cognitive processes of their relationship looking at them through the perspective of resource depletion [53]. Based on the COR theory [60], the study has built and tested a conceptual model that gives an understanding on how despotic leadership leads to incivility at workplace. The results of H1 show that despotic leadership also has a positive relationship with workplace incivility.

The results of this finding were reported in the responses of 381 employees in the higher education sector in Islamabad/Rawalpindi. It conforms to the theoretical frame work of this study, and advances a growing body of knowledge about the complex relation between despotic leadership and people’s self-regulatory resources [140]. Employees who face mistreatment from leaders often rely on their self-regulatory resources to manage stress, regulate emotions, and maintain professional conduct [141]. Under despotic leadership, distributive justice and equity in workload, which are essential for managing workplace challenges, tend to decrease. Employees are then forced to invest their resources into understanding their supervisor’s intentions, controlling frustration, and managing their emotional responses.

The observed positive relationship suggests that as employees face higher levels of despotic leadership, their finite self-regulatory resources are more likely to be depleted. This depletion reflects the psychological strain of dealing with the challenges caused by despotic leadership. Our results supported past literature, which suggests that under despotic leadership, employees feel frustrated due to unrealistic demands and excessive workload, ultimately leading to workplace incivility.

The second hypothesis of our study, which states that” Despotic leadership has a positive and significant impact on workload,” is also supported. This finding is consistent with past studies, which suggest that under despotic leadership, the leader often places unrealistic demands on employees, leading to increased workload. Despotic leaders tend to be authoritarian and controlling, usually micromanaging and demanding excessive output from their subordinates. When the workload increases, employees engage in uncivil behavior. Chaudhary and Islam [10] This could be due to several reasons: employees may be unable to leave work behind and focus on other activities, and there is injustice in resource allocations and workload equity. They may experience high frustration levels due to excessive and unrealistic demands from their leader, which is supported by our findings.

The findings of hypothesis are supported by previous studies, highlighting that such continuous strain can eventually lead to burnout, a state of emotional, physical, and mental exhaustion caused by prolonged stress. Employees who manage increasing workloads may also encounter workplace incivility, hostile behavior, or mistreatment. This combination of burnout and incivility can exacerbate employees’ challenges, undermining their well-being and job satisfaction. Increased work demands consume more of an employee’s time and energy and have limited resources. As demands increase, employees are unable to fulfil their other activities, feeling depleted in personal resources such as psychological, physical, and social benefits resources that help them succeed. As a result, they may exhibit negative behavior, leading to workplace incivility. In comparison [14] reported that the presence of supportive leadership styles, such as transformational leadership, could significantly mitigate the negative effects of increased workload, reducing the likelihood of incivility. This highlights that the relationship between workload and incivility is not universal, but contingent on the leadership context a point that our findings reinforce.

Organizations are focusing on providing distributive justice and ensuring equity in workload distribution. When employees are given too much work by their leaders throughout the day, they feel pressured. This pressure makes it difficult for them to focus on home activities because most of their energy is spent dealing with work demands, leaving little for recovery activities [142]. As a result, employees use their resources to cope with the workload, leaving fewer resources for recovery [143]. Our findings aligned with previous research, which shows that despotic leadership often places unrealistic demands on employees, leading to an increased workload.

The third Hypothesis in Our Study, which stated that” Workload has a positive and significant impact on workplace incivility,” was supported by our findings. Our results are consistent with previous research, showing that increasing workloads force employees to put extra physical and emotional effort to meet deadlines and complete tasks. Over time, this pressure can lead to fatigue, with employees feeling extremely exhausted. For instance, Marcionetti and Castelli [91] found that many employees reported high levels of physical tiredness after trying to manage workplace demands. This exhaustion drains their energy, affecting their ability to perform well in the following days.

If this stress continues, it can lead to burnout, which is feeling very tired mentally, physically, and emotionally because of long-term pressure. As employees try to manage larger workloads, they may also experience workplace incivility, which includes negative behaviors or mistreatment at work. Incivility can worsen employees’ challenges and damage workplace environments. Our results aligned with past literature that employees face more stress and pressure when workloads increase, leading to frustration and exhaustion [144]. This makes it harder for them to stay calm and respectful, resulting in rude or unprofessional behavior. The more workload they have, the more likely they experience stress, negatively affecting their interactions with colleagues. Therefore, higher workloads can lead to more workplace incivility.

The Fourth Hypothesis in Our Research, “Workload mediates the relationship between despotic leadership and workplace incivility,” is accepted. It supports prior academic research that suggested that workload mediates the DL and WPI relationship, and increased workload under despotic leadership leads to stress. Additionally, employees who work under despotic leaders often have a heavier workload, which causes more stress [145]. Employees in such situations may feel tired all the time and emotionally drained. As a result, they are more likely to behave poorly at work, which can harm the organization. Despotic leaders contribute to stress, leading to more workplace incivility. The results support the premise of Conservation of Resources (COR) theory, which suggests that when employees experience a lack of personal resources, which can be of psychological, material, and social benefits, frustration builds up. This frustration can manifest in negative behaviors, ultimately leading to workplace incivility. Leadership has always been a critical determinant of organizational performance, which is why it has received significant attention. However, the dark side of leadership has been largely neglected, particularly in the higher education sector of South Asian countries [8]. Our results, in contrast to some previous research, suggest that low job control in the academic institutions studied further strengthens the mediating role of workload. This divergence reinforces the importance of contextual variables in shaping the pathways from leadership to employee outcomes, providing a more comprehensive understanding of these dynamics.

Finally, the study highlights the negative effects of despotic leadership and its role in triggering negative employee behavior. Despotic leaders create feelings of discomfort, anxiety, and depression, which can lead employees to engage in workplace incivility, either consciously or subconsciously. Employees who exhibit uncivil behavior often feel that their leaders exploit them for personal gain, causing frustration that eventually results in incivility. It’s also crucial to note that despotic leadership not only lowers employee performance Khan, Gan [146] but also negatively affects mental health. Despotism creates a sense of psychological contract violation, leading to stress, which in turn triggers bad behavior and incivility in the workplace. As a result, overall workplace incivility increases.

The final hypothesis of the study suggests that,” Distributive justice moderates the relationship between workload and workplace incivility such that the relationship will be weakened when distributive justice is high and vice versa,” is supported. The findings align with previous studies, which suggest that a high level of distributive justice within organizations reduces workplace incivility. This is consistent with the research of [147], which demonstrate that distributive justice negatively impacts workplace incivility.

Employees who feel that the resources provided by management meet their expectations and needs are less likely to consider leaving the organization. Therefore, management must ensure fair resource allocation based on employees’ responsibilities, which helps maintain a positive work environment. When rewards are perceived as fair, employees feel happier and more comfortable. Conversely, a despotic leadership, which is marked by authoritarianism and dominating behavior often forces the employees to work under excess and irrelevant tasks whereby they add on to their burden. This results in low perception of distributive justice because of leader’s despotic behavior which drains the employees’ psychological resource. Employees are likely to portray negative behaviors when psychologically damaged which leads to incivility at work [148] Absence of distributive justice, despotic leadership worsens workplace incivility. When the employees experience an unfair allocation of resources and workloads, the negative impact of despotic leadership is exaggerated, thus, causing more stress and more workplace incivility. Unequal resource allocation may such lead to stress which will ultimately lead to incivility in the workplace [149].

In summary, our study not only confirms several established findings in the literature but also provides new insights into how contextual factors such as sector, culture, and institutional support can influence the dynamics between despotic leadership, workload, and workplace incivility. Where our results diverge from previous studies, these differences appear to be rooted in the specific context of higher education in Pakistan, underscoring the value of context-sensitive research in organisational behaviour [18].

### Theoretical implications

The proposed hypotheses have several theoretical implications in the context of higher education institutions in Islamabad/Rawalpindi. The first hypothesis H1 indicates that academic institutions led by despotic and authoritarian leaders may lead to more disrespectful behaviour and conflicts within the faculty. Taking a controlling and unsympathetic approach, a despotic leader can make the academic setting unhealthy and raise workplace incivility. The H2 hypothesis points out that leaders with dictatorial behaviour make faculty members in higher education institutions work harder by setting unrealistic goals and high standards. This creates more stress, more anger, and less time for personal life, which has a negative impact on work conditions. According to H3, promoting the idea that too much work increases stress and dissatisfaction can result in bad behaviour at work. Too much work may make employees feel frustrated and annoyed, causing them to act in an uncivil way towards each other. H4 points out that when despotism increases, employees’ workloads go up, which leads to more incivility. In effect, as leaders are more dominant, employees face extra work, which eventually brings about incivility in the work environment. Lastly, H5 states that when it comes to resource allocations and workload distribution, the distributive justice can reduce some negative impact of excessive workload on workplace incivility. If the workload distribution is perceived to be equitable by the employees, then they may refrain from uncivil behaviors even during high work demands under despotic leadership and create a good working environment in the context of higher education, On balance, these hypotheses form a complete picture of the different dynamics involved in the despotic leadership, workload and workplace incivility in the peculiar cultural and organizational setting of higher education establishments in Islamabad/Rawalpindi. It is based on COR theory that this study rests. COR theory, which is used in the case of the Higher education sector in Rawalpindi Islamabad, explains the proposed relationships on a theoretical basis. It implies that, individuals should attempt to safeguard their resources such as time, energy and support. Despotic leadership forces employees to take on excessive tasks, draining their resources. This creates a sense of unfairness in the distribution of workload, leading to a loss of psychological resources. When these resources are depleted, employees may act out, resulting in workplace incivility. This supports H1, as despotic leadership creates an environment where incivility increases. COR theory also supports H2, showing that despotic leaders place excessive workload demands on employees, negatively affecting their mental well-being. The depletion of resources leads to negative behaviors like incivility. H3 is aligned with the theory, as excessive workload from despotic leadership contributes to stress and incivility.H4 suggests that workload mediates the relationship between despotic leadership and workplace incivility, showing how increased workload depletes resources and employees show bad behavior can lead to workplace incivility to negative behaviors. Finally, H5 highlights the role of distributive justice when employees feel resources are fairly distributed, the equity in workload, and lessening workplace incivility context of higher education institutions in Islamabad/Rawalpindi.

### Implications for practice

New research has pointed out that incivility in workplaces is of particular concern in higher education in Pakistan. 71 percent of university faculty have experienced significant levels of incivility, according to [59]. The problems that often arise for leaders are still often not examined. Because of the strong power-distance culture in Pakistan, despotic leadership is typical in higher education circles. It reveals that despotic leadership affects work environments and presses for actions to correct this. It is clear from the evidence that when leaders are despotic, resources are wasted. Leadership behaviours that are negative should be met by organisational intervention [4,150]. Nonetheless, pinpointing despotic management is hard, because workers might be afraid of suffering any consequences [3,67]. Groups must work to correct their hiring and career-advancement procedures to keep despotic leaders from rising [151]. It’s important for employees to have a simple way to report problems anonymously [152]. It has also been found that authoritarian leadership overloads workers, resulting in uncivil behaviour at work (WPI). Here, DJ is very important. If workloads are distributed equally, organisations can lower the impact of those, who act in a despotic way. HR has the duty to encourage fairness and cheque up on occasional complaints of biassed treatment. Training staff in how to be respectful and polite has shown to decrease workplace incivility [153]. Additionally, reducing stress in the workplace through positive support can help minimise the bad effects of despotic leadership [154–156]. Staff members who believe that resources and duties are divided fairly are able to handle pressure and continue to develop a positive work atmosphere. Anyone who feels mistreated requires additional help, but those treated a little better are better able to cope with stress. It leads us to ask how hiring practices can block despotic leaders and how DJ can guard against workplace incivility.

### Limitations

Our study has some limitations. First, we collected data exclusively from the higher education sector in Islamabad and Rawalpindi; therefore, the results apply only to this sector in these two cities. Future studies could include data from other cities in Pakistan to determine if different working environments yield different results. Since we focused only on Islamabad and Rawalpindi, the findings may not apply to other areas. Future research could examine other sectors and the global context. Secondly, we used a method called convenience sampling to collect our data. This means we selected people who were easily available to provide the data, so the results might not represent everyone. As a result, it limits the generalizability of the findings to a larger group. Thirdly, employees were often busy with their workload and were not always willing to provide data properly. Furthermore, many employees responded without properly reading the questions, resulting in low generalizability. Fourthly, we had limited time to complete our research, so we could only collect data from two cities. Research takes a lot of time, and due to resource constraints, it wasn’t possible to visit other cities in person to gather more data. If the sample size needs to be larger, more time would be required. Fifthly, due to time constraints the study used a cross-sectional design, which means we collected data at only one point in time. This makes it hard to see changes or developments over time. Because of this, the findings might not show the full picture of the relationships being studied. Lastly, we used the SPSS tool to analyse our data. In future studies, researchers might consider using more advanced tools like Mplus, SmartPLS or R Studio to handle complex models.

### Future directions

First, The Future research can address an examination of the impact of despotic leadership and workplace incivility within diverse organisational settings, industries, and cultures to determine possible dissimilarities and cooperabilities in results. Second, valuable insights into the long term would come from longitudinal studies, effects of despotic leadership on workplace incivility, which covers both immediate and delayed consequences to organisational culture. Third, Additionally, other factors such as cognitive dissonance and neurotic tendencies may be used as mediators in the relationship between despotic leadership and incivility at the workplace, suggesting that workload could be used as a mediator. Fourth, future research could explore other possible moderators of this relationship, such as organisational climate, leader-member exchange (LMX), or individual characteristics such as resilience and emotional intelligence, and psychological capital including hope, optimism, and self-efficacy in order to obtain more comprehensive set of insights on dynamics involved. Fifth, scholars could put efforts in designing interventions and strategies that can moderately soften the negative effects of despotic leadership for instance through leadership training programmes, strategies to end conflicts in organisations and organisational practices that enhance respect and civility. Sixth, for a more comprehensive understanding and the potential for universal applicability, future research should prioritise cross-speciality comparisons. This strategy can help determine whether the identified patterns are universally applicable across various medical disciplines or are unique to the speciality under investigation. Seventh, Reassuring the audience of the quality of our work, conducting longitudinal studies is crucial for gaining a comprehensive understanding of how variables and outcomes evolve. These studies are instrumental in identifying causal relationships and long-term effects that may not be captured in a cross-sectional design, thereby ensuring the thoroughness of our research.

Lastly, this research used non-probability and convenience sampling techniques in order to collect data from the higher education sector in Islamabad and Rawalpindi to test the research hypotheses. For further research, it is suggested that the probability sampling techniques, for instance, random or cluster technique, are to be used to procure data from different fields in Pakistan. This strategy would help make comparisons more effective and testing the research hypotheses more effective.

## Conclusion

Our present study on research has revealed how despotic leadership affects incivility in the higher education of Islamabad-Rawalpindi. It also examined how workload is a mediator and distributive justice as a moderator in the relationship between DL and WPI. Through examining these factors, the study gives new insights on workplace dynamics in despotic leadership, a pertinent issue in the current organisational frameworks.

The first one objective was to bring out how despotic leadership has a negative impact of workplace incivility. This research contributes to the existing knowledge in the field of leadership in the higher education sector and delivers practical implications for organisations. HR should cheque the complaints made regarding unfair treatments. Training programmes should aim at providing an equal workload distribution and distributive justice hence lessening workplace incivility and developing positive organisational culture. Such measures can promote healthier and more productive work environment.

Although, the area of study was narrowed to the higher education sector in Islamabad and Rawalpindi; to thus limit the findings within this framework, further studies should extend to regions other than this for higher generalizability and validity.
